# Reprocessed Materials Used in Rotationally Moulded Sandwich Structures for Enhancing Environmental Sustainability: Low-Velocity Impact and Flexure-after-Impact Responses

**DOI:** 10.3390/ma15186491

**Published:** 2022-09-19

**Authors:** Abu Saifullah, Pappu Radhakrishnan, Lei Wang, Burhan Saeed, Forkan Sarker, Hom N. Dhakal

**Affiliations:** 1 Advanced Polymers and Composites (APC) Research Group, School of Mechanical and Design Engineering, University of Portsmouth, Portsmouth PO1 3DJ, UK; 2 Matrix Polymers, Northampton NN3 9AG, UK; 3 Department of Textile Engineering, Dhaka University of Engineering & Technology, Gazipur 1707, Bangladesh

**Keywords:** rotational moulding, sandwich, environmental sustainability, reprocessed materials, low-velocity impact (LVI), flexure-after-impact (FAI), impact damage

## Abstract

In the rotational moulding industry, non-used, scrap, and waste purge materials have tremendous potential to be reprocessed and applied in skin-foam-skin sandwich structures to replace and reduce the use of virgin polymers. This approach not only encourages the re-use of these waste materials but also significantly contributes to reduce environmental impacts associated with the use of virgin polymers in this sector. The demand of rotationally moulded sandwich structures is rapidly increasing in automotive, marine, and storage tanks, where investigating their impact and after-impact responses are crucial. Hence, this study investigated the low-velocity impact (LVI) and flexure-after-impact (FAI) responses of rotationally moulded sandwich structures manufactured using reprocessed materials. Results obtained from LVI induced damage at two different incident energy levels (15 J, 30 J), and the residual flexural strength of impacted structures evaluated by three-points bending tests were compared with non-reprocessed sandwich structures (virgin materials). The impact damage progression mechanism was characterized using the X-ray micro-computer-tomography technique. Reprocessed sandwiches demonstrated 91% and 66% post-impact residual strength at 15 J and 30 J respectively, while for non-reprocessed sandwiches, these values were calculated as 93% and 88%. Although reprocessed sandwich structures showed a lower performance over non-reprocessed sandwiches, they have a strong potential to be used in sandwich structures for various applications.

## 1. Introduction

Rotational moulding is a unique and the most suitable manufacturing process to produce complex-shaped, large, one-piece, hollow plastic structures [1,2,3]. In this process, both single and multiple-layered plastic products can be produced easily, and they are considered competitive to produce in other plastic moulding processes, such as injection and compression mouldings. One of the examples of multiple-layered rotationally moulded plastics is skin-foam-skin sandwich structures, which are increasingly used in many structural and semi-structural load-bearing applications, such as automotive panels and bumpers [4], boats, kayaks, canoes, and large storage tanks (oil and water) [5], because of their high bending strength, light weight, excellent skin-foam layer interfacial properties, acoustic and thermal insulation, etc. [6,7].

Recently, in general, conventional synthetic plastic industries are facing huge challenges to tackle environmental pollution problems and maintain sustainability throughout the whole sector using more recyclable and bio-degradable materials, energy efficient manufacturing processes, and cleaner production initiatives. Likewise, plastic industries also need to address and plan immediately so that the industries could be in line with the target of achieving a net zero emissions goal, defined by COP26, by the middle of the current century. Without an exception, the rotational moulding industry is also facing same challenges for moving forward towards achieving sustainability. 

Rotationally moulded products are normally used multiple times in their 20–30–year life span, and mostly thermoplastic materials are used in manufacturing them. Theoretically, they must be fully recyclable. Therefore, recycling the used rotomoulded products at the end of their lives and using them again in new product cycles is a lucrative solution to deal with the plastic wastes in this sector. However, in reality, some major problems, such as the large size of the used products, small number of the recovered products, complex recovery process, and collection cost, have made this solution very difficult to achieve with the existing industrial supply chain set-up [8]. Considering this problem, using roto-moulded reprocessed polymers and/or commercially available recycled post-consumer household products or post-industrial plastics and their mixtures from non-rotomoulded products (injection, blow moulded, pipe, household, constructions, etc.) could be potential solutions to reduce and replace the use of virgin plastic materials and hence minimise harmful environmental emissions in this sector.

A limited number of works have been found in the literature wherein recycled single-source and post-consumer wastes (mainly polyethylene (PE)) were blended with roto-mould grade virgin PE at different blend ratio combinations for rotationally moulded foam [9] and single-layer plastics materials [10,11,12]. From these works, it is clear that the recycled materials could potentially be used in the rotational moulding process, while manufacturing process optimization (optimized blend ratio, powder size, viscosity, etc.) is very much needed for an improved result in terms of easier moulding and aesthetic and mechanical properties. 

Reprocessed material is another option to be used in rotational moulding, as discussed before. Reprocessed materials include rotationally moulded off-cuts, scarp and non-used parts, and waste purge materials. They can be reground for polymer powder generation and used again in the rotational moulding process [8]. The reported work by Pick et al. investigated the reprocessed (reground non-used product) rotationally moulded materials for single-layer plastics and compared them with fully virgin and blended virgin/recycled materials [13]. Reprocessed materials showed very similar and comparable mechanical properties to the virgin materials. The use of reprocessed materials can be further extended to the multilayer or sandwich structures, as they are capable of maintaining a good mechanical property. A 5000 L capacity storage tank made of rotationally moulded multi-layered structure requires more than 300 kg of virgin polymers [8]. Hence, it can be easily understood that replacing even a layer of sandwich structures with the reprocessed material will save huge quantity of virgin polymers, which in turn will reduce emissions and have positive environmental impacts. 

In the above-mentioned load-carrying applications of rotationally moulded sandwich structures, impact incidents from collisions with debris or other structures and tool drops during maintenance are considered very likely to happen [5,6]. Damage occurring in any impact event can adversely affect the structural integrity and reduce the overall load-carrying performance of sandwich structures. Because of this, it is necessary to investigate the impact and post-impact residual strength behavior of sandwich structures before using them confidently in any structurally demanding applications. Low-velocity impact (LVI) properties analyses presented by various studies for rotationally moulded sandwich structures, including the effects of various impact energy levels [14], different skin/core thickness combinations [6], and different foam densities, have highlighted that understanding impact-induced damages is important [5]. In real-life situations, LVI events can take place for sandwich structures in different ways with different incident impact energies, which require to generate the impact velocity up to 10 m/s, according to Richardson and Wisheart [15]. In addition, the post-impact residual strength of impacted sandwich structures was also studied by employing the flexure-after-impact (FAI) technique using the three-point bending test arrangement [5]. Although the post-impact residual strength can be measured with other techniques, such as compression-after impact (CAI) [16] or tension after-impact (TAI) after impact [17], FAI is considered as a straightforward test comparatively. In addition, this is fully relevant to many applications where the bending load (combination of compression and tension) is mainly applied on the structures [18]. 

There are hardly any reported works available on the manufacturing of rotationally moulded sandwich structures using reprocessed materials and investigation of their impact induced damage and post-impact behaviours. This is considered as a clear gap in the literature and also rotational moulding industry for promoting the use of reprocessed materials in sandwich structures for various applications. Therefore, in this work, we manufactured sandwich structures with non-reprocessed (virgin materials) and reprocessed materials and conducted their low-velocity impact, damage progression, and post-impact residual strength tests using flexure-after-impact (FAI) set-up. An in-depth comparison was made in terms of impact and post-impact residual properties between reprocessed and non-reprocessed sandwich structures to understand the behaviour of reprocessed sandwich structures for the first time in this study so that their uses can be extended in load-carrying applications confidently in the rotational moulding sector. This investigation will make a significant contribution to save a huge amount of virgin polymer materials in sandwich structures through replacing them with reprocessed materials and ultimately promote environmental sustainability and promote circularity in this sector. 

## 2. Materials and Methods

### 2.1. Materials and Manufacturing of Rotationally Moulded Sandwich Structures

Sandwich structures manufactured with virgin and reprocessed materials are termed in this work as non-reprocessed and reprocessed sandwich, respectively. Both sandwich types were manufactured at the rotational moulding facility of Matrix Polymers Ltd., Northampton, UK, using a Carousel type machine (Ferry Industries, Stow, OH, USA). Commercially available rotomould grade PE and reprocessed PE and PE foam core materials were used to manufacture sandwich samples. For the PE foam core layers, an ADC (Azo dicarbonamide) blowing agent was blended in the main PE polymer matrix. Material details for the manufacturing of sandwich structures are provided in Table 1. The only difference between these two types of sandwich structures was having reprocessed PE materials as the lower skin in the reprocessed sandwich, while normal virgin PE material was used for the non-reprocessed sandwich. The lower skin plays a vital role for the impact damage initiation and propagation, as identified in previous studies [5,6], and therefore, it was aimed to understand the impact and post-impact resistances of reprocessed materials used in the lower skin of reprocessed sandwich structures. This understanding will contribute to develop reprocessed sandwich structures fully based on reprocessed materials in any other future work.

**Figure 1:**
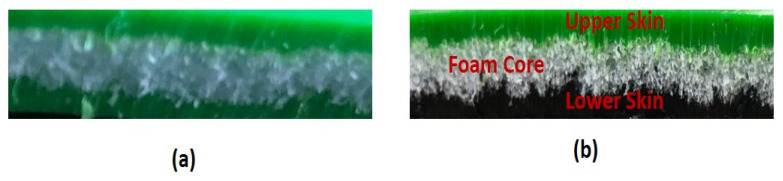
Rotationally moulded (**a**) non-reprocessed and (**b**) reprocessed sandwich structures.

Similar manufacturing conditions were used for manufacturing of both types of sandwiches. The upper skin layer material was added first inside the mould (Ferry Industries, Stow, OH, USA). and heated up to 135 °C by placing the mould in the heating oven (Ferry Industries, Stow, OH, USA); after that, the mould was taken out from the oven to add the foam core materials and heated back in the oven up to 160 °C; finally, the lower skin layer materials was added following the same method and heated up to 195 °C to fully expand the foam core layer along with the proper formation of the upper and lower skin layers. Examples of sandwich samples are given in Figure 1.

### 2.2. Impact Test (LVI)

**Figure 2:**
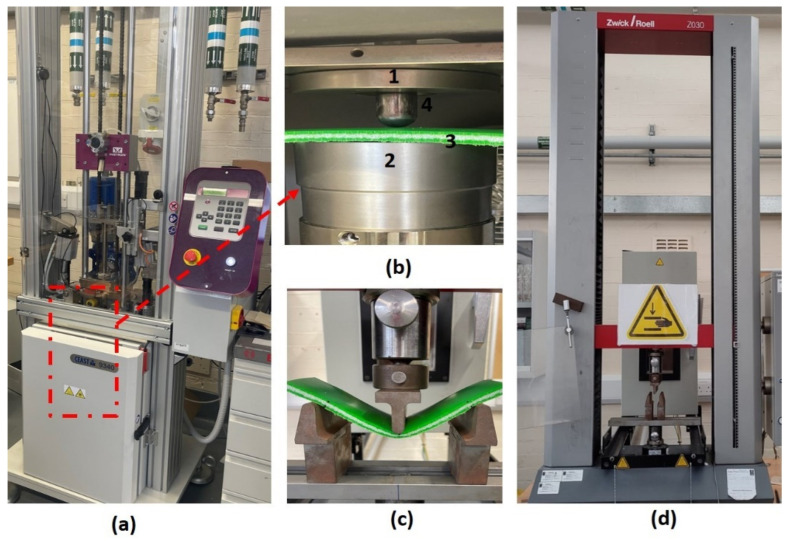
Testing equipment and set-up; (**a**) low-velocity impact testing machine, (**b**) impact testing sample holder arrangement (1 and 2—upper and lower parts of the sample holder, respectively; 3—sandwich specimen; 4—impactor nose), (**c**) three-point bending set-up with sandwich specimen for flexure-after-impact (FAI) test, and (**d**) FAI test machine.

An instrumented falling-weight impact tester (INSTRON CEAST 9340, Norwood, MA, USA) was used to conduct the low-velocity impact test (LVI) of sandwich structures (Figure 2). ASTM-D3763-02 standard was followed to perform the testing at 15 J and 30 J incident energy levels. The hemispherical impactor nose had a diameter of 19 mm. Initially, sandwich panels were cut from the 300 mm cube moulded products, and these panels were utilized to obtain the testing specimens with dimensions of 200 mm length, 75 mm width, and 8 mm thickness. LVI tests were carried out by placing and clamping test specimens in the sample holder of the impact machine. The impactor hit the specimens on upper skins. For each sandwich type, three specimens were tested at each impact energy level. From the impact testing, force-time, force-deflection, and absorbed energy-time data were analysed. 

### 2.3. Post-Impact Residual Strength Measurement with Flexure-after-Impact Test (FAI)

A three-point-bending test set-up was used for measuring the FAI properties of impacted and non-impacted reprocessed and non-reprocessed sandwiches in order to measure, compare, and analyse their post-impact residual strength and behaviours. FAI tests were performed following the ISO-178 [19] test standard in a Zwick/Roell, (Stourbridge, UK) universal tester with a 10 kN load cell, support span length of 130 mm, and 3 mm/min displacement rate up-to a total deflection of 50 mm (Figure 2). Three specimens were tested for both impacted and non-impacted specimens of reprocessed and non-reprocessed sandwich types. The test specimen deflection was measured according to the ISO-178 test standard requirements from the test machine’s crosshead displacement, applying the compliance correction. Residual strength values were calculated at flexural impact force points ($F_{m}$) using the following equation:

(1) $$\mathrm{Residual}\;\mathrm{Strength}=\;\frac{3F_{m}L}{2bh^{2}}$$

Here, *L* = support span length; *b* = specimen width; *h* = specimen thickness.

### 2.4. Impact Damage Characterisation

Initially, impact damage images at upper and lower skins were taken and analysed using a digital camera (Canon EOS 700D, Uxbridge, UK) to identify the overall failure modes.

As an advanced technique, the X-ray micro-computer tomography (µ-CT) was employed with aNikon XTH 225 scanner (Leuven, Belgium) to scan the damage area of the impacted sandwich structures. The X-ray u-CT technique helped to investigate and identify the impact damage initiation and progression mechanism clearly within the impacted sandwich structures. The scanning was set to 145 kV and 147 µA with a Perkins Elmer flat panel detector (Santa Clara, CA, USA). The voxel size was 0.084 mm and a total of 1562 images were taken to produce 3D volume for the image analysis using VGStudios Maxver 2.0.5 software.

## 3. Results and Discussion

### 3.1. Low-Velocity Impact (LVI) Properties

**Figure 3:**
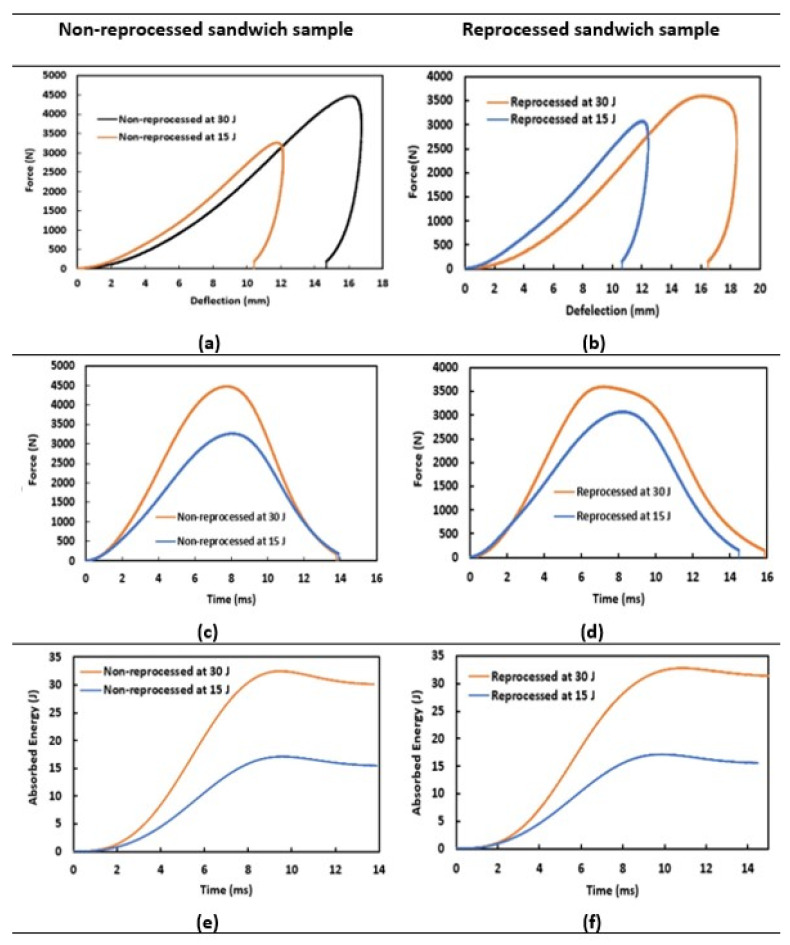
Low-velocity impact properties. (**a**) Force-deflection, (**c**) force-time, and (**e**) absorbed energy-time of non-reprocessed and (**b**) force-deflection, (**d**) force-time, and (**f**) absorbed energy-time of reprocessed sandwich structures.

Figure 3 and Table 2 provide LVI properties (force-time, force-deflection, and absorbed energy-time data) of impact-tested non-reprocessed and reprocessed sandwich structures at 15 J and 30 J impact energy levels. In force-deflection curves (Figure 3a,b), overall, a higher impact force and deflection value were measured for 30 J impact energy compared to 15 J for all tested specimens, as expected. In addition, the impact force values up to around 12 mm deflection point in the ascending curves at the 30 J impact energy were slightly lower compared to the corresponding values at the 15 J energy level. This is attributed to the overall higher deflection and deformation or damage creation in both types of tested sandwich structures at the 30 J impact energy level. It was also clearly found the that reprocessed sandwich showed lower peak impact forces with higher deflection values compared to non-reprocessed sandwich in both energy levels, which is related to a better stiffness and impact resistance of non-reprocessed sandwich structures. 

Force-deflection curves are important to understand the damage mechanisms of impacted sandwich specimens [20]. In general, for all the impact events, the impactor hit the test specimens, and hence, the impact force was found to increase with specimen deflection up to the peak impact force point (loading part in the curve), wherein a smooth transition or a slight drop in the force values were observed before starting the decrease of the impact force down to the zero value (unloading part) as the impactor was rebounded from the tested specimens. For any of the impact events, the impactor’s full penetration through the sandwich structures was not noticed because the unloading parts of the force-deflection curves ultimately reached back to the X-axis. At the 15 J impact energy level, force-deflection curves were smooth at the peak impact force point for both sandwich specimens, as no clear crack was seen. In addition, the impact resistance behaviour was very similar since this impact energy was not able to create any significant deformation in any of the tested sandwich structures. For the 30 J energy level, reprocessed sandwich specimens demonstrated a slight drop in the peak impact force, followed up with a plateau region, and these are linked up with damage initiation and progression behaviour, respectively, while the non-reprocessed sandwich did not exhibit any of these features. These behaviours were also found to be obvious since a clear damage was observed in the reprocessed sandwich specimens, which is provided and described in the damage analysis section.

Reprocessed sandwich structures also revealed lower peak impact force values compared to non-reprocessed sandwich specimens in force-time (Figure 3c,d) curves, and the peak impact forces showed an increase in their values with tested energy levels without showing any surprise. The slight drop (damage initiation) with a subsequent plateau region (damage progression) in the peak impact force of reprocessed sandwich specimens tested at 30 J energy was prominently seen in the force-time graphs (Figure 3d), which was found absent for non-reprocessed sandwich specimens (Figure 3c), as discussed above. Absorbed energy-time graphs are also included in Figure 3e,f for non-reprocessed and processed sandwiches. Absorbed energy graphs are presented for the whole impact event duration so that the tested sandwich structures’ energy absorption behaviour can be understood for the whole impact duration and also analysed in relation to their force-time and force-displacement graphs. It was seen that the area under the curves were increasing for the 30 J compared to 15 J energy level. The impacted energies (15 J and 30 J) were not sufficient to penetrate the tested specimens. Due to that, both sandwich structures absorbed the full impacted energy during the tests at 15 J and 30 J, with a small indication of elastic energy (impactor rebounding), which can be seen in the graphs.

From the impact properties details, it is evident that the reprocessed sandwich structures have less impact resistance than that of the non-reprocessed sandwiches. Although they showed almost similar impact resistances at 15 J impact energy, reprocessed sandwiches became cracked at 30 J. For the peak impact force (Table 2), non-reprocessed sandwich specimens showed 3263 N and 4462 N at 15 J and 30 J energy levels, whereas reprocessed sandwich specimens were reported as 3070 N and 3588 N, respectively, for these energy levels.

### 3.2. Analysis of Imapct Damage Mechanisms 

**Figure 4:**
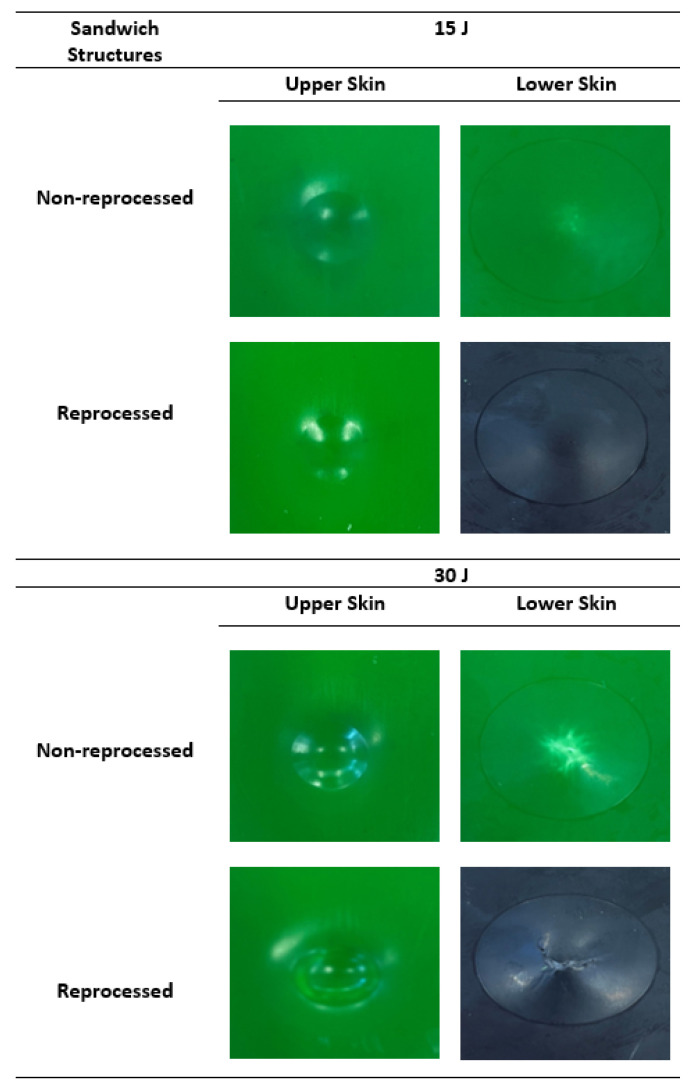
Impact damage at lower and upper skins of non-reprocessed and reprocessed sandwich structures at 15 J and 30 J impact energy levels.

Figure 4 provides the outer and lower skin images of non-reprocessed and reprocessed sandwich specimens after impact at 15 J and 30 J to understand the initial and overall damage mechanisms. The indentation depth was seen on upper skins for both sandwich structures, and the indentation depth became deeper with increasing impact energy levels, as expected. At 15 J impact energy, the non-reprocessed sandwich showed protrusions with white stretch marks at the lower skins. Similarly, reprocessed sandwich specimens showed protrusions but no white stretch marks, and this could be due to the use of black colour pigment for moulding the lower skins of reprocessed sandwiches, which ultimately made the white stretches invisible. The protrusion area was increased with the impact energy level. The white stretch marks became larger and more obvious at 30 J without any crack in the lower skins for non-reprocessed specimens. Reprocessed sandwich specimens clearly showed a large crack in the lower skin at 30 J. This supports the earlier observation in Section 3.1 about a slight drop in the peak impact force of force-deflection or force-time graphs of reprocessed sandwich structures.

**Figure 5:**
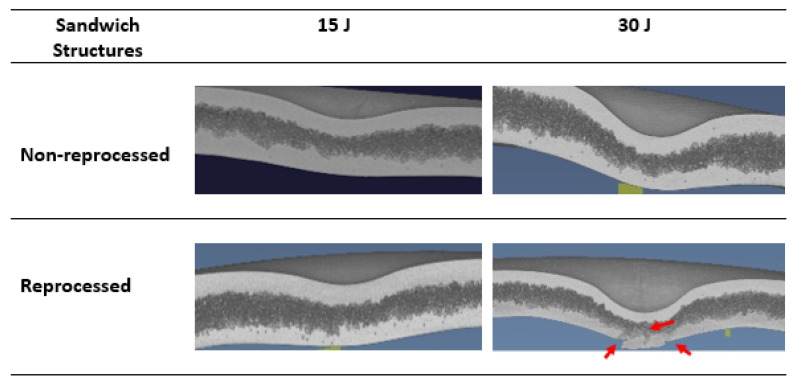
Section views of the X-ray damage images taken at the middle of the damage area of impacted non-reprocessed and reprocessed sandwich specimens at 15 J and 30 J impact energy levels. Red arrow marks indicate the cracks.

**Figure 6:**
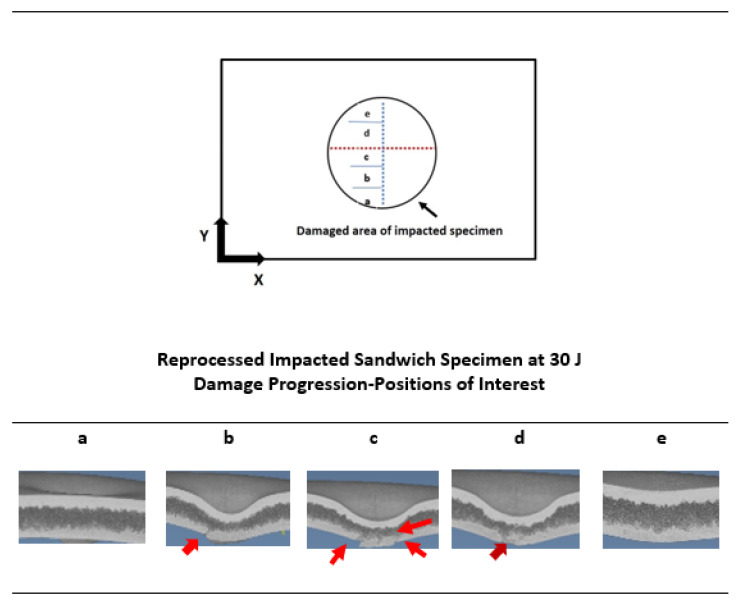
Progression of impact damage (a–e positions of interest) for reprocessed sandwich structure at 30 J. Red arrows indicate the cracks. Positions of interest (a–e) represent the different points of the damaged area of impacted specimen, shown in the upper image of this figure.

**Figure 7:**
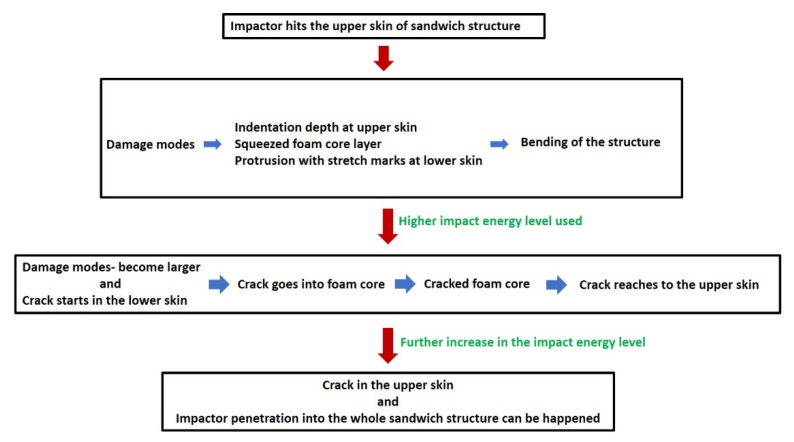
Damage progression mechanism of rotationally moulded sandwich structure during a low-velocity impact (LVI) event.

Comparing damage at 15 J and 30 J impact energies of both sandwich types, it is clear that the crack starts from the lower skins, and this observation was also found in previous studies [5,6]. When the impactor attacks and transfers impact energy to the upper skins in an impact event, the compression force is applied, and the indentation depth is created. This ultimately leads to the bending of whole structure and particularly the tension or stretching force in the lower skins. As a result of this, white stretch marks are clearly formed in the lower skins. Due to the bending, the foam core layer is squeezed along its thickness reduction. For the further increase of impact energy level, the stretching or tension force is reached in their upper limit, and hence, they cannot be accommodated further, which ultimately creates a crack in lower skins. To investigate this damage mechanism in details, the X-ray µ-CT scanning technique was used to scan the inside of the damage area. This X-ray scanning normally supplies the damage images in cross-sections of different layers that help to understand the damage progression behaviour of the tested sandwich structures [21]. The X-ray-µ-CT-scanned section-view images at the middle of the damage area of impacted non-reprocessed and reprocessed sandwich specimens are given in Figure 5. At 15 J, indentation depth at upper skins, core layer thickness reduction, and lower skin protrusion damage modes were visible for both sandwich structures. At 30 J, these damage modes became larger for non-reprocessed sandwich structures. However, for reprocessed sandwich structures, the crack clearly started from the lower skins and propagated into the foam layer. For both types of sandwich structures at 15 J and 30 J energy levels, the buckling of the whole structure was visible. The buckling event can happened to a sandwich structure that has a tough foam core layer, and the foam core layer can provide good support to the skin layers during the impact [22]. The X-ray-scanned images at different positions of the damage area of reprocessed specimens at 30 J are provided in Figure 6 since the crack was formed only in reprocessed sandwich structures at 30 J. At positions a and e, no crack was seen, as they were at the very edges of the damage area. If positions b, c, and d are considered, the damage mechanism can be easily understood. The crack was initiated from the lower skin and propagated into the foam layer, which also cracked, and finally reached to the upper skin. The upper skin did not show any crack because the impact energy at 30 J was not enough to create any crack in the upper skin except the indentation depth. Based on these observations, the steps in the damage progression mechanism are drawn in Figure 7. Advantageously, no delamination was observed at the skin–foam interfaces. This gives a unique strength point of rotationally moulded sandwich structures since normally, delamination is a major concern of any skin-foam-skin sandwich structures and reduces the mechanical load bearing capacity of the structure significantly after impact. Additionally, all the damage modes are visible and easy to detect. Hence, there is no concern for the bare visible damage in the structure, which propagates very rapidly in the structure from a small damage and provides no chance of taking preventive measures before the catastrophic failure of the whole sandwich structure. The observed damage mechanisms will be supportive to develop a mathematical theoretical analysis using damage mechanics theories in the future, which is not covered in this study.

### 3.3. Flexure-after-Impact (FAI) Properties for Post-Impact Residual Strength Analysis 

**Figure 8:**
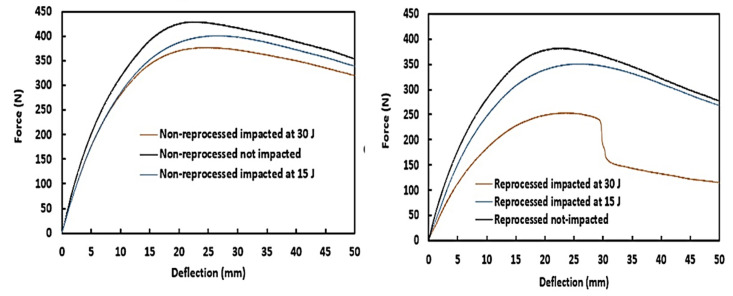
Force-deflection curves at FAI tests of non-reprocessed and reprocessed sandwich structures using three-point-bending test set-up.

**Figure 9:**
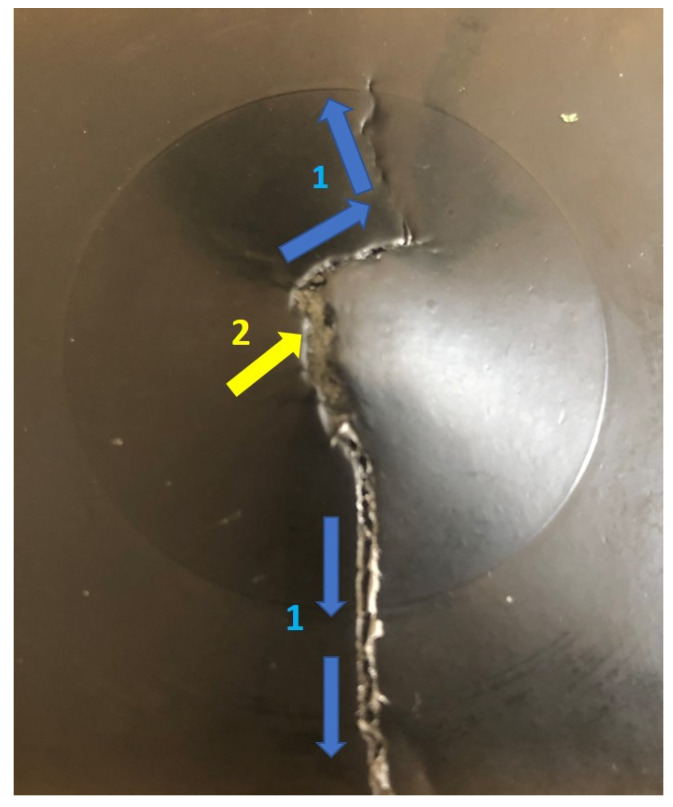
Lower skin damage progression of 30 J impacted reprocessed sandwich specimen during FAI test. 1 with blue mark—damage progression directions; 2 with yellow mark—cracked foam core.

Force-deflection curves of FAI tested non-reprocessed and reprocessed sandwich structures are given in Figure 8. Flexural test provides very crucial mechanical responses of any structures to the external loads [23] combining tensile and compressive forces. FAI properties are also included in Table 3 for both not-impacted and impacted types of sandwich structures. In Figure 8, the reprocessed sandwich specimens show two different types of curves. The first type of curve was demonstrated with reprocessed not-impacted and impacted at 15 J specimens where the flexural force was seen to increase up to the peak impact force with a subsequent smooth transition to the decrease of force until a total of 50 mm overall structure deflection was reached. Reprocessed specimen impacted at 30 J exhibited the second type of curve since a drop in the flexural force was seen just after the peak impact force, followed by a slow decrease of force until the 50 mm deflection point was reached. This occurred due to the damage progression from the impact damage origin to the edge of the tested specimen and ultimate failure of lower skin. For all tested (not-impacted and impacted at 15 J and 30 J) non-reprocessed sandwich specimens, only one type of force-deflection curve was observed; flexural force values were reached up to the peak impact force and decreased to the 50 mm deflection point without any disruption. Because of this, as expected, no significant changes in the impacted damage of the non-reprocessed specimens were found during FAI tests. In contrast, Figure 9 shows the lower skin failure during the FAI test of reprocessed specimen impact at 30 J, which also supports the drop in the peak impact force of its force-deflection curve. Damage was progressed from the origin of impact crack to the edge of the specimen due to applied tensile forces in the lower skin during the FAI test, and hence, lower skin was failed. The broken foam core layer was also clearly noticed. Although the lower skin was failed, the reprocessed sandwich structure was still able to carry on the load, as the broken foam core and indented upper skin were supporting the load. In Figure 9, only reprocessed specimen impacted at 30 J was presented, as no other specimens either from non-reprocessed or reprocessed sandwich types showed any significant FAI damage progression. 

From the Table 3 FAI properties data, it was found that flexural modulus values were reduced for reprocessed sandwich specimens with impact energy levels compared to their not-impacted counterparts. Likewise, flexural peak force and force at the 50 mm deflection point also followed the similar trend. The reduction of FAI properties’ values for 30 J impacted specimens was significant due to the lower skin failure. Non-reprocessed sandwich structures had higher FAI properties compared to reprocessed sandwich structures. Non-reprocessed sandwich specimens also showed a decreasing pattern for all FAI properties (modulus, peak force, and force at 50 mm deflection point) with impact energy levels although the reduction was not very significant for 30 J impacted specimens over 15 J impacted specimens. Residual strength is also provided in Table 3 for both sandwich structures, which was calculated from flexural peak forces according to the flexural stress calculation equation mentioned in Equation 1 and presented in the ISO-178 testing standard. This was used to calculate the normalized residual strength, which is expressed as the ratio of residual strength of each sandwich structure specimens at different energy levels to the residual strength of their not-impacted specimens. As expected, the non-reprocessed sandwich structures showed higher normalized residual strength and also followed the same decreasing pattern with impact energies. From normalized residual strength values, it is understood that non-reprocessed sandwich structures had 93% and 88% load-carrying capacity of their original specimens after impacted at 15 J and 30 J, respectively. These values were found as 91% and 66%, respectively, for reprocessed sandwich specimens. The lowest normalized strength was found for reprocessed sandwich specimens impacted at 30 J. 

Based on above mentioned results and discussion, it is very clear that reprocessed sandwich structure had lower impact and FAI properties with a quicker damage progression mechanism compared to non-reprocessed sandwich structures, particularly at 15 J and 30 J impact energy levels. Moreover, reprocessed sandwiches also showed lower flexural modulus and flexural peak force for the original specimens (not-impacted). These observed differences between reprocessed and non-reprocessed sandwich structures resulted because of the use of reprocessed plastic layer in the lower skin of the reprocessed sandwich structures. Reprocessing involves some additional processing steps (regrinding of waste materials, scraps, re-rotational moulding, etc.) that are not applicable for virgin materials. Although the reprocessed materials are not used in practical product life conditions, the additional processing steps may degrade the polymer structure. This can lead to a decrease in plastic extensibility and an increase in brittleness. Generally, reprocessing of plastics is accompanied by a reduction in thermal and mechanical properties [24,25], which are more prominent in impact tests [25]. In an impact event, polymer chains in a plastic material face a dynamic loading condition, and to withstand this load, polymer chains need to have flexible structures for the absorption of a sudden, intense load. This reasoning is also true for our work. The used reprocessing materials in the reprocessed sandwich specimens reduced the energy absorption and ductility properties of the whole structure, and therefore, a lower impact resistance and post-impact residual strength was noticed for the reprocessed structures in this work. Although this comparative analysis evidences a reduced performance of reprocessed sandwich structures, they have a strong ability to carry out any impact load less than 30 J with a good flexural and post-impact residual strength properties. Therefore, it is believed that this work will promote the use of reprocessed materials in rotationally moulded sandwich structures, and this needs to be carried out with a careful analysis and selection of various applications considering the range of required impact, flexural, or post-impact residual strength conditions. 

## 4. Conclusions

Low-velocity impact (LVI) and flexure-after-impact (FAI) responses at 15 J and 30 J impact energy levels were investigated for reprocessed rotationally moulded sandwich structures, and they were compared with non-reprocessed sandwich structures. For manufacturing of reprocessed sandwich structures, only at lower skins were reprocessed materials used. Reprocessed sandwich structures demonstrated a lower impact performance, with a crack formation at 30 J impact test, while non-reprocessed sandwich structures were able to resist all impact energies tested in this study. Both sandwich structures showed upper skin indentation depth, squeezed foam core, protruded lower skin with stretch marks, no skin/core interfacial delamination, and catastrophic failure as damage modes. Reprocessed sandwich structures also had lower FAI properties over non-reprocessed sandwich structures, as expected, and the impact crack that occurred at 30 J impacted specimen progressed, and finally, the lower skin failed during the FAI test. It was found that reprocessed sandwich structures retained 91% and 66% residual strength after impact at 15 J and 30 J, respectively, while these values were found as 93% and 88% for non-reprocessed sandwich structures compared to their not-impacted specimens. The reprocessing can cause degradation in polymer chains that leads to lower impact and mechanical properties of reprocessed sandwich structures. It is evidenced that reprocessed materials can be used in rotational moulded sandwich structures more favourably at the lower skins. The performance of reprocessed materials in the upper skin and foam core requires further understanding and will be investigated in a future study. The findings of this study will be helpful to extend the use of reprocessed materials in sandwich structures for the rotational moulding industry that will certainly reduce virgin materials consumption significantly and hence utilize material resources effectively with less environmental emissions and a relatively improved environmental sustainability and circularity. 

## Figures and Tables

**Table 1:** Materials and sandwich structure details ^1^.

Non-Reprocessed Sandwich				Reprocessed Sandwich		
	Materials	MFI (g/10 min)	Density (g/cm^3^)	Materials	MFI (g/10 min)	Density (g/cm^3^)
Upper skin	PE	3.50	0.949	PE	3.50	0.949
Foam Core	PE closed cell foam	3.50	0.310	PE closed cell foam	3.50	0.310
Lower skin	Same as upper skin			**Reprocessed PE**	7	0.935
Thickness	2 mm upper skin, 4 mm foam core, and 2 mm lower skin			2 mm upper skin, 4 mm foam core, and 2 mm lower skin		

^1^ Materials data were collected the supplier (Matrix Polymers, UK).

**Table 2:** Low-velocity impact (LVI) properties of sandwich samples.

	Low-Velocity Impact (LVI) Properties			
	Non-Reprocessed Sandwich		Reprocessed Sandwich	
	Impact Energy			
	15 J	30 J	15 J	30 J
Peak Impact Force (N)	3263 (±66)	4462 (±109)	3070 (±42)	3588 (±58)
Maximum Deflection (mm)	12.08 (±0.20)	16.70 (±0.27)	12.40 (±0.15)	18.34 (±0.20)
Impact Time (ms)	13.90 (±0.11)	13.80 (±0.20)	14.48 (±0.10)	15.85 (±0.12)
Absorbed Energy (J)	15	30	15	30

**Table 3:** Flexure-after-impact (FAI) properties of sandwich structures.

	Flexure-after-Impact (FAI) Properties					
	Non-Reprocessed Sandwich			Reprocessed Sandwich		
	Impact Energy					
	Not-impacted	15 J	30 J	Not-impacted	15 J	30 J
Flexural Modulus (MPa)	653 (±10)	540 (±10)	530 (±7)	530 (±8)	450 (±5)	300 (±8)
Flexural Peak Force (N)	428 (±6.89)	400 (±10.24)	377 (±15.22)	381 (±2.38)	350 (±2.04)	252 (±1.15)
Force at 50 mm Deflection (N)	353 (±0.43)	338 (±4.84)	317 (±11.04)	276 (±1.38)	267 (±1.34)	115 (±2.52)
Residual Strength (MPa)	17.38	16.25	15.31	15.47	14.21	10.23
Normalised Strength	1	0.93	0.88	1	0.91	0.66

## Data Availability

Not applicable.
